# Systems Pharmacology-Based Approach of Connecting Disease Genes in Genome-Wide Association Studies with Traditional Chinese Medicine

**DOI:** 10.1155/2018/7697356

**Published:** 2018-03-22

**Authors:** Jihye Kim, Minjae Yoo, Jimin Shin, Hyunmin Kim, Jaewoo Kang, Aik Choon Tan

**Affiliations:** ^1^Translational Bioinformatics and Cancer Systems Biology Laboratory, Division of Medical Oncology, Department of Medicine, School of Medicine, University of Colorado Anschutz Medical Campus, Aurora, CO 80045, USA; ^2^Department of Computer Science and Engineering, Interdisciplinary Graduate Program in Bioinformatics, Korea University, Seoul, Republic of Korea

## Abstract

Traditional Chinese medicine (TCM) originated in ancient China has been practiced over thousands of years for treating various symptoms and diseases. However, the molecular mechanisms of TCM in treating these diseases remain unknown. In this study, we employ a systems pharmacology-based approach for connecting GWAS diseases with TCM for potential drug repurposing and repositioning. We studied 102 TCM components and their target genes by analyzing microarray gene expression experiments. We constructed disease-gene networks from 2558 GWAS studies. We applied a systems pharmacology approach to prioritize disease-target genes. Using this bioinformatics approach, we analyzed 14,713 GWAS disease-TCM-target gene pairs and identified 115 disease-gene pairs with *q* value < 0.2. We validated several of these GWAS disease-TCM-target gene pairs with literature evidence, demonstrating that this computational approach could reveal novel indications for TCM. We also develop TCM-Disease web application to facilitate the traditional Chinese medicine drug repurposing efforts. Systems pharmacology is a promising approach for connecting GWAS diseases with TCM for potential drug repurposing and repositioning. The computational approaches described in this study could be easily expandable to other disease-gene network analysis.

## 1. Introduction

Traditional Chinese medicine (TCM) has been practiced over thousands of years in China and other Asian countries for treating various symptoms and diseases [1]. The drug development process for the modern (or Western) medicine is usually initiated by developing small molecules that target a specific biological target or pathway [2]. In this drug development process, it is much easier to study the mechanism of action (MoA) of these small molecules in a context-specific disease state. For example, vemurafenib was developed to specifically inhibit BRAF p.V600E-mutant cancer cells [3]. Clinically, vemurafenib has demonstrated efficacy in treating BRAF-mutant melanoma patients, eventually received Food and Drug Administration (FDA) approval in 2011 for the treatment of late-stage melanoma [4]. Due to the known MoA (e.g., binding to BRAF mutants and inhibiting the downstream signaling pathway) and the target population (e.g., melanoma with BRAF p.V600E), it is much easier to develop predictive biomarker (e.g., BRAF p.V600E) for personalize treatment using modern drug [4]. However, it remains a challenge to elucidate the molecular mechanisms of a particular TCM in inhibiting biological process in a context-specific disease state because of the “multicomponent, multitarget” nature of TCM.

The polypharmacology property of TCM could be exploited for drug repurposing and repositioning. For example, artemisinin, an active component extracted from *Artemisia annua* L. in the early 1970s in China, has been the “magic bullet” for malaria therapy [5]. In late last year, through a high-throughput chemical biology screen, a group of researchers identified artemisinin can induce pancreatic alpha cells to transdifferentiate into insulin producing beta-like cells [6]. This finding suggests that artemisinin could be repurposed as type 1 diabetes therapy. It also opens up other opportunities to use modern technologies to reveal the unexpected interactions between TCM and diseases for drug repurposing.

With the advancement of high-throughput genotyping technologies (e.g., microarray and sequencing), it is now possible to perform genome-wide association studies (GWAS) to identify candidate genes associated with various complex diseases [7]. One of the challenges in GWAS is to translate the candidate genes into clinic for disease management and treatment. Similarly, microarray and sequencing technologies have been applied in chemical biology research to deconvolute target genes and to elucidate molecular mechanisms [8, 9]. These modern biotechnologies open up new research avenues for the investigation of TCM in a wide range of diseases. To gain insights, novel computational algorithms and bioinformatics methods are needed to analyze and interpret these massive “omics” data. Systems pharmacology is a promising research approach in translational medicine that integrates computational and experimental methods to elucidate, validate, and apply new pharmacological concepts to the development and use of small molecule and biologic drugs [10, 11]. Approaches in systems pharmacology can provide a scientifically rational for defining optimal multidrug regimens, identifying responsive patient populations, identifying translational biomarkers, drug repurposing, and designing clinical trials [10–13].

**Figure 1:**
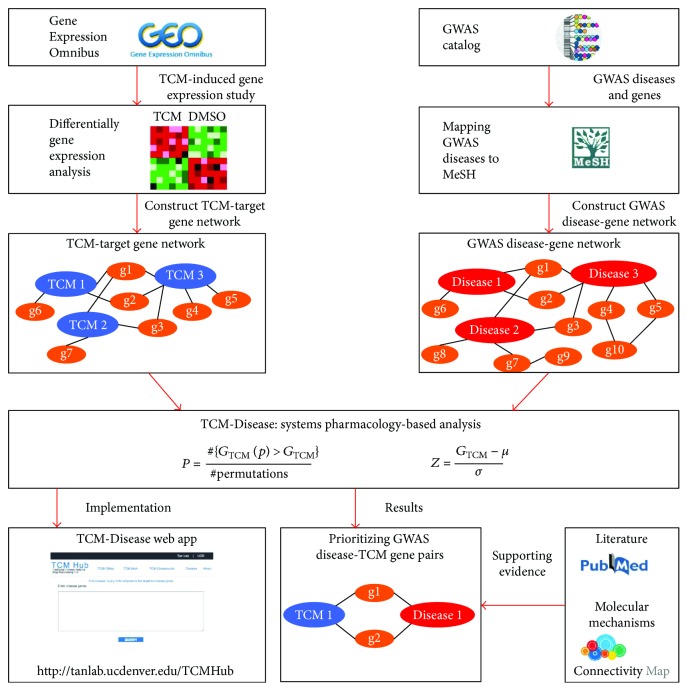
Overall research strategy of systems pharmacology-based analysis of connecting GWAS diseases to TCM-target genes.

In this study, we employed systems pharmacology-based approach of connecting GWAS disease genes with traditional Chinese medicine as drug repurposing and repositioning strategy. We obtained TCM-induced gene expression study from the Gene Expression Omnibus and performed differentially expressed gene analysis to define TCM-target genes. We constructed a drug-gene network based on the TCM-target genes. We downloaded candidate genes associated with GWAS diseases from GWAS catalog [7] and constructed the GWAS disease-gene network for this study. By using a systems pharmacology-based analysis, we prioritized GWAS disease-TCM gene pairs from the two networks. We performed literature and molecular mechanisms evidence by querying PubMed and the Connectivity Map to support the GWAS disease-TCM gene pairs. We also implemented TCM-Disease web app for users to query new connections between disease and TCM-target genes. Our overall research strategy of this study is illustrated in Figure 1.

## 2. Materials and Methods

### 2.1. TCM Gene Expression Analysis

We downloaded the raw microarray data of 102 TCM component-induced gene expression studies from the Gene Expression Omnibus (GSE85871) [14]. These TCM molecules are active ingredients in Chinese herbs and TCM. Briefly, TCM molecules (either 1 *μ*M or 10 *μ*M) and DMSO were treated on MCF7 breast cancer cell line, and total RNA was extracted and subjected for Affymetrix HG U133A 2.0 microarray gene expression profiling in duplicate. Raw data were downloaded and normalized by Robust Multiarray Average using Affymetrix Power Tools. For data analysis, we collapsed the probe sets representing the same gene using the maximum expression value of these probe sets to improve power to detect any changes in different isoforms. Gene expression values from the duplicates were averaged, and fold change of 1.5 was used as the thresholds to select differentially expressed genes for a particular TCM compound against DMSO. We used the list of differentially expressed genes as the gene signatures as the TCM-target genes. The list of 102 TCM components is listed in Supplementary Table 1.

### 2.2. Genome-Wide Association Studies Disease Genes

We obtained the latest collection of genome-wide association studies (GWAS) data from the NHGRI-EBI GWAS catalog of published GWAS (https://www.ebi.ac.uk/gwas/home, data version: v1.0, gwas-catalog_v1.0-associations_e89_r2017–06-19.tsv) [7]. The GWAS catalog of GWAS contains 1987 diseases/traits collected from 2558 studies. We used the genes associated with the diseases in this study.

### 2.3. Mapping Disease Terms to MeSH

GWAS diseases obtained from the NHGRI-EBI GWAS catalog were mapped to the Medical Subject Headings (MeSH) terms. MeSH is the NIH/NLM/NCBI controlled vocabulary thesaurus used for indexing articles for PubMed, and it follows a hierarchical tree structure for indexing these terms. We mapped the GWAS disease names using the MeSH Browser (https://meshb.nlm.nih.gov/search), such that we could group similar diseases using the MeSH terminology tree. For this study, we focused on diseases that mapped to the MeSH terms in the “Disease [C]” category, using the third term level in the MeSH tree [CX.X.X]. We grouped all the genes associated with the same MeSH term and used as the genes associated with that disease term.

### 2.4. Protein-Protein Interactions Network

To construct the GWAS disease-gene network, we queried STRING to retrieve additional genes (proteins) associated with a particular set of disease genes [15]. We restrict the protein-protein interactions from STRING and only include not more than 100 addition genes that have high-confidence interactions score (score > 0.9). We build the GWAS disease-gene network using the resultant protein-protein interactions.

### 2.5. Statistical Framework for Prioritizing TCM-Disease Connection

To prioritize TCM-Disease connection, we performed a permutation test to compute the statistical significance of a TCM to be prioritized for treating a specific disease [16]. Our hypothesis is that the polypharmacology nature of TCM has a potential to treat a particular disease if its targets are associated with that disease-gene network. We performed a permutation based testing as below to obtain the nominal *p* value (*P*) similar to the approach of Connectivity Map [8, 9]:

(1) $$\begin{array}{c} P=\frac{\#\left\{G_{TCM}\left(p\right)>G_{TCM}\right\}}{\#permutations}, \end{array}$$

where *G*_TCM_ represents the number of GWAS disease genes targeted by a TCM. *G*_TCM_(*p*) represents a permutation that GWAS disease genes were targeted by a TCM. We performed 100,000 permutations by randomly selecting the same number of genes in a GWAS disease from a genome-wide scale (we used 20,462 genes as the number of genes) in this study. A nominal *p* value (*P*) is calculated for each TCM by counting the number of permutations that have *G*_TCM_(*p*) greater than *G*_TCM_, divided by the total number of permutations. Next, we performed Benjamini-Hochberg multiple testing correction method to obtain the adjusted *p* values (*q* values) for the TCM-GWAS disease genes.

To compare the TCM-target genes against each GWAS disease, we calculated a *Z*-score for each disease as:

(2) $$\begin{array}{c} Z=\frac{G_{TCM}-\mu }{\sigma \;}, \end{array}$$

where *G*_TCM_ denotes the number of GWAS disease genes targeted by a TCM, *μ* denotes the mean number of GWAS disease genes targeted by a given TCM, and *σ* represents the standard deviation during the 100,000 permutations. The permutation test and *Z*-score calculation are inspired by the approach used in [16].

### 2.6. TCM-Disease Web Application Implementation

We developed TCM-Disease, the statistical framework described in this study, as a user-friendly web application. The website is implemented using Python 2.7.9, Python-CGI script deployed in a Mac OSX Server. The web application is part of the traditional Chinese medicine drug repurposing hub (TCM hub), which is freely accessible at http://tanlab.ucdenver.edu/TCMHub.

### 2.7. Network Visualization

To visualize the connections between GWAS disease-TCM-target genes, we utilized the Cytoscape network visualization tool (version 3.5.1) [17, 18].

### 2.8. Connectivity Map Analysis

We utilized the latest Connectivity Map (CMap) CLUE web application (https://clue.io) resource for identifying MoA for these TCM components [8, 9]. The latest CMap resource contains more than 1.3 million gene expression signatures that were collected from genetic or small molecule perturbations on a panel of cell lines. The CMap version (Data version 1.0.1.1. and Software Version 1.0.1.8) contains 2836, 3799, and 2160 signatures for compounds, gene knockdown, and gene overexpression, respectively. We queried the top 150 upregulated and downregulated genes for each TCM against CLUE App to retrieve the connected signatures as potential molecular mechanisms of TCM components. The results of the Connectivity Map for these 102 TCM components are available in TCM hub (http://tanlab.ucdenver.edu/TCMHub).

## 3. Results

### 3.1. Construction of the TCM-Target Gene Network

We constructed the TCM-target gene network from the analyzing microarray gene expression profiles of 102 TCM components. The final TCM-target gene network consists of 43,839 interaction pairs, which connect 102 TCM components and 7380 target genes.  Table 1 summarizes the statistics of the TCM-target gene network. From the TCM-target gene network, it suggests that TCM could be repurposed for various diseases as indicated by the polypharmacology properties of these compounds.

### 3.2. Construction of the GWAS Disease-Gene Network

To study the disease-gene interactions, we constructed the disease-gene network from 2558 GWAS studies. We grouped the GWAS diseases using MeSH terms and only focused on the diseases in the MeSH “Disease [C]” vocabulary term in this study. The final GWAS disease-gene network consists of 39,262 interaction pairs, which connect 293 diseases and 7417 disease genes.  Table 1 summarizes the statistics of the GWAS disease-gene network.

### 3.3. Systems Pharmacology-Based Analysis of Connecting GWAS Disease-TCM-Target Genes

**Figure 2:**
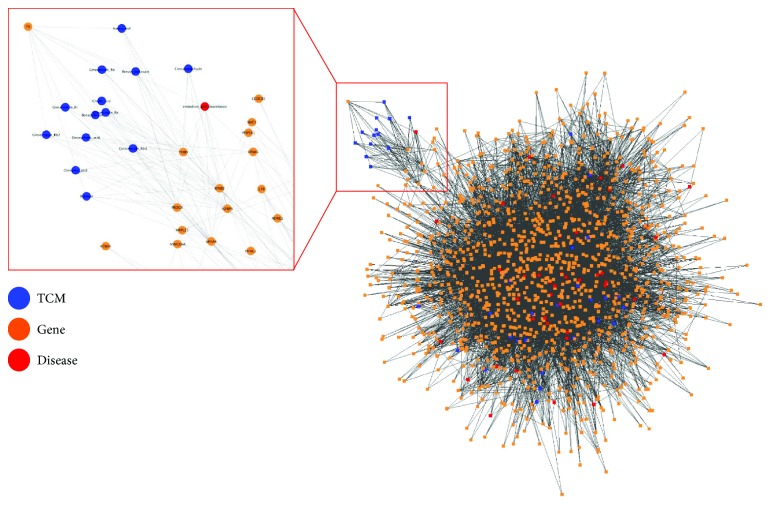
GWAS disease-TCM gene network. This is the GWAS disease-TCM gene network.

**Figure 3:**
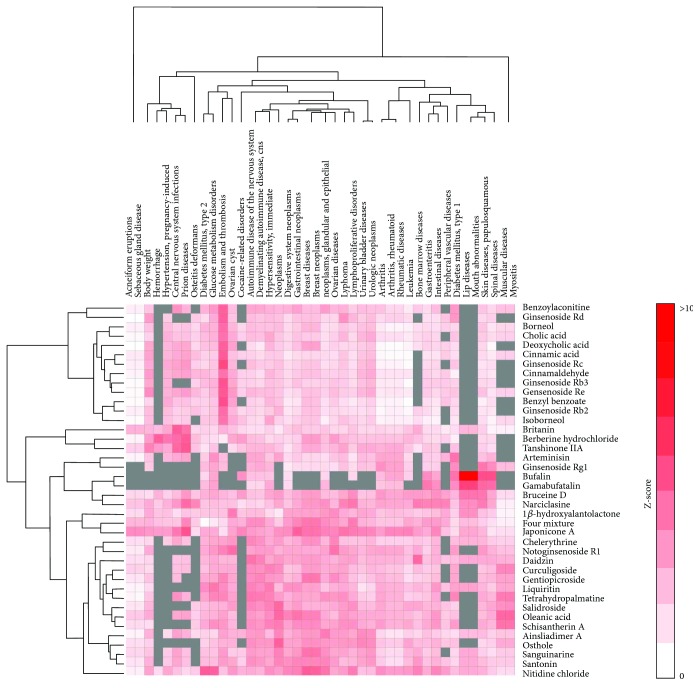
Heatmap showing the statistical significance GWAS disease-TCM gene pairs by *Z*-score.

We performed the statistical systems pharmacology analysis to determine the connections between GWAS disease genes and TCM-target genes. We performed 14,713 connections between GWAS disease-TCM-target genes comparison using the described statistical framework as described in the Method section. Here, we used the *q* value < 0.2 as the threshold and identified 115 GWAS disease-TCM-target gene pairs from the analysis.  Figure 2 shows the GWAS disease-TCM gene network. This list of GWAS disease-TCM-target gene pairs consists of 40 TCM and 42 GWAS diseases as summarized in Table 2.  Figure 3 illustrates the heatmap of the 40 TCM and 42 GWAS diseases.

### 3.4. Case Study of GWAS Disease-TCM Gene Pairs

We illustrate the following GWAS disease-TCM gene pairs identified from the bioinformatics analysis and provide experimental validations supported by published literature. We also queried the TCM gene expression signatures to the latest version of Connectivity Map for potential mechanism of action (MoA) evidence of the TCM against GWAS diseases.

#### 3.4.1. Japonicone A as Lymphoma Treatment

**Figure 4:**
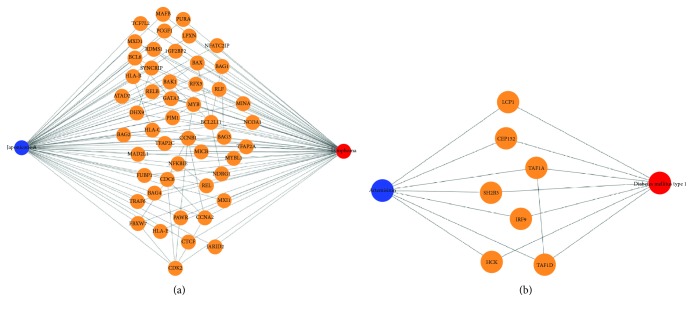
Example of GWAS disease-TCM gene networks. (a) Japonicone A as lymphoma treatment. (b) Artemisinin as type 1 diabetes mellitus treatment.

We identified japonicone A as a potential treatment for lymphoma (*q* value = 0.06) (Figure 4(a)). Japonicone A is a natural product extracted from the aerial part of *Inula japonica* Thunb (Asteraceae family) [19] and has been demonstrated to possess activities in anti-inflammatory, antidiabetes, and anticancer [19, 20]. By querying the TCM hub, japonicone A is connected with NF-*κ*B pathway inhibitors as one of the mechanisms of action (MoA). Interestingly, japonicone A has been recently demonstrated to inhibit the growth of Burkitt lymphoma via inhibiting the NF-*κ*B pathway [21]. This provides experimental support of japonicone A that could be repurposed as the lymphoma treatment.

#### 3.4.2. Artemisinin as Type 1 Diabetes Mellitus Treatment

From the systems pharmacology-based analysis, we identified artemisinin, an antimalaria drug, that is connected with type 1 diabetes mellitus (*q* value = 0.11) (Figure 4(b)). Artemisinin is a compound extracted from *Artemisia annua* L. (Chinese name: qinghao). The discovery of artemisinin by Tu Youyou and her colleague has been the breakthrough therapy for malaria [5], and this discovery has led to the 2015 Nobel Prize of Physiology or Medicine awarded Tu Youyou. Notably, in a recent study, it has been demosntrated that treating antimalarial drug artemisinin in pancreatic alpha cells could transform into functional beta-like cells through enhanced GABA signaling [6]. From the gene expression analysis, GABA is one of the top overexpressed genes after artemisinin treatment. This demonstrates that this systems pharmacology approach could identify artemisinin as drug repurposed for diabetes mellitus treatment.

#### 3.4.3. Sanguinarine as Breast Cancer Treatment

Another GWAS disease-TCM gene pair identified from this study is sanguinarine as breast cancer treatment (*q* value = 0.07). Sanguinarine is a compound extract from bloodroot (*Sanguinaria canadensis*) and has been used to treat different diseases [22]. Recent study has demonstrated that sanguinarine has anticancer therapy in inhibiting cell migration and the induction of apoptosis in basal-like breast cancer. The authors also identified that the sanguinarine is a potent dihydrofolate reductase (DHFR) inhibitor [23]. This provides evidence that sanguinarine could be used as anticancer drug for the treatment of basal-like breast cancer through systems pharmacology analysis.

#### 3.4.4. Berberine Hydrochloride as Preeclampsia Treatment

From the GWAS disease-TCM genes analysis, we identified berberine hydrochloride as a potential pregnancy-induced hypertension (preeclampsia) (*q* value = 0.13). Berberine hydrochloride is an extract from the Chinese goldthread *Coptis chinensis*. This TCM compound has been shown to antioxidant, anti-inflammatory, and antimicrobial activities [24, 25]. It has been recently demonstrated that berberine hydrochloride could alleviate preeclampsia by regulating IL2/IL10 and BCL2/BAX expressions in preeclampsia rat models [26]. This warrants further investigation of using berberine hydrochloride for the treatment of preeclampsia.

#### 3.4.5. Tanshinone IIA as Obesity Treatment

Finally, the systems pharmacology analysis identifies tanshinone IIA as treatment for obesity (*q* value = 0.19). Tanshinone IIA, a commonly used TCM for antioxidant and anti-inflammatory, is a compound extracted from *Salvia miltiorrhiza* (Chinese: Danshen) [27]. It has been recently demonstrated that tanshinone IIA could be used for obesity treatment through peroxisome proliferator-activated receptor (PPAR) gamma antagonism [28]. This provides a drug repurposing of tanshinone IIA for obesity treatment.

Taken together, we showed that the GWAS disease-TCM gene pairs identified in this study could be validated by literature evidence. Furthermore, the MoA of these TCM against the GWAS disease could be supported by Connectivity Map analysis.

### 3.5. TCM-Disease Web Application

**Figure 5:**
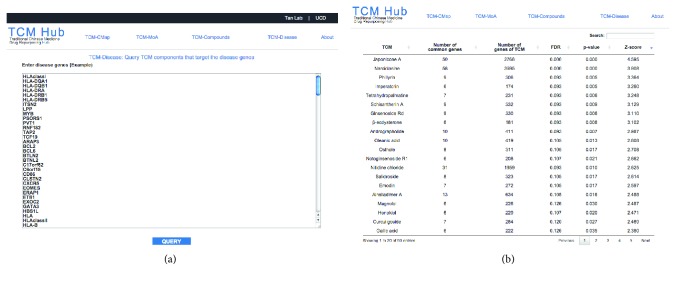
Screenshot of the TCM-Disease web application. (a) A list of disease-related genes are entering into the query box. (b) The web application performs the systems pharmacology permutation test and *Z*-score and returns the TCM component sorted by *p* value. This example is querying the lymphoma disease genes, and japonicone A is returned as the top TCM connected to this disease.

To facilitate the translation of connecting TCM-target genes with GWAS disease genes, we implemented the statistical framework as TCM-Disease web application. For the TCM-Disease web application, users will submit the list of GWAS disease genes and perform the statistical test for identifying disease-TCM gene pairs. The web application returns the list of disease-TCM gene pairs (currently the database consists of 102 TCM) ranked by the permutation *p* values and *Z*-scores.  Figure 5 shows the screenshot of the TCM-Disease web application.

## 4. Discussion

TCM has been successfully applied to treat various diseases for the past several thousand years, due to its polypharmacology nature. In this study, we performed a systems pharmacology-based approach to connect GWAS disease genes with TCM-target genes. We derived 102 TCM components and their target genes by analyzing microarray gene expression experiments. We constructed disease-gene networks from 2558 GWAS studies. We applied a systems pharmacology framework to prioritize disease-target genes as potential repurposing and repositioning TCM for these diseases. Using this bioinformatics approach, we analyzed 14,713 GWAS disease-TCM-target gene pairs and identified 115 disease-gene pairs with *q* value < 0.2. We validated several of these GWAS disease-TCM-target gene pairs with literature evidence, demonstrating that this computational approach could reveal novel indications for TCM.

Systems pharmacology and network biology approaches have been applied to study disease-gene interactions [16, 29–34]. For example, Fang et al. employed systems pharmacology-based approach to prioritize natural products that target somatic-mutated genes in cancer [16]. They introduced the statistical framework applied in this study, and they demonstrated that by using this approach they could identify clinically actionable alterations in cancer using natural products [16]. Kim et al. performed a systematic analysis of structural similarities between traditional oriental medicine (TOM) compounds and human metabolites for studying the mechanisms of action and suggest approaches to reduce toxicity [35]. They identified that TOM compounds could provide the complementary action, neutralizing action, facilitating action, and pharmacokinetic potentiation in 38 different synergistic combinations [35]. This provides an opportunity to use TCM/TOM in combination with other modern therapeutics. All of these studies rely on using systems pharmacology or network biology approaches to elucidate the disease-TCM gene interactions. This supports the use of systems pharmacology-based approach in the current study of identifying GWAS disease-TCM-target gene pairs.

TCM-Disease web application is part of the TCM hub—a bioinformatics resource for traditional Chinese medicine drug repurposing study (http://tanlab.ucdenver.edu/TCMHub). TCM hub currently consists of four web applications: (1) TCM-CMap, (2) TCM-MoA, (3) TCM-Compounds, and (4) TCM-Disease. TCM-CMap is the implementation of the “connectivity map” concept based on 102 TCM compounds. The TCM-CMap web app allows users to connect a gene signature (up and down genes) to one of the 102 TCM compounds in the reference database. TCM-MoA web app allows users to search the molecular mechanisms of TCM compounds. TCM-Compounds web app provides the CLUE connection results for the 102 TCM compounds. The CLUE connection results include connecting the TCM compound gene signatures to 2836, 3799, and 2160 signatures for compounds (CP), gene knockdown (KD), and gene overexpression (OE), respectively. TCM-Disease web app allows users to identify disease-TCM gene pairs for potential drug repurposing and repositioning. We believe that TCM hub represents a unique bioinformatics resource for in silico study of traditional Chinese medicine.

There are several limitations of the current GWAS disease-TCM gene networks study. First, the number of TCM is limited; currently, this study is only assessing 102 TCM components based on gene expression changes, which may limit the coverage of TCM-target gene networks. Second, the number of diseases investigated in the current study is limited to 293 GWAS diseases, which may limit the coverage of disease-gene networks. Lastly, our current validation of the associations of GWAS disease-TCM genes is based on literature evidence with experimental results. To address these limitations, as part of our future work, we plan to increase the number of TCM components by mining Gene Expression Omnibus (GEO) for additional TCM microarray studies. We will complement the gene expression signatures with TCM-target genes by mining established drug-target databases such as TCMID [36], TCM-MeSH [37], PharmDB-K [38], or DSigDB [39]. By integrating the gene expression targets and manually curated targets, we can expand the coverage of TCM-target gene networks. In parallel, we plan to expand the disease-gene networks by using data curated from DisGeNet [40], currently contains 561,119 gene-disease associations and more than 9000 diseases. By constructing the disease-gene network from this large database, it will increase the coverage of the diseases and potentially increased the value in revealing novel disease-TCM genes.

## 5. Conclusions

In summary, we present a systems pharmacology-based approach for connecting GWAS diseases with TCM for potential drug repurposing and repositioning. We employed several unique bioinformatics and computational approaches to identify and prioritize GWAS disease-TCM gene pairs. The computational approaches described in this study could be easily expandable to other disease-gene network analysis. We also develop TCM-Disease web application to facilitate the traditional Chinese medicine drug repurposing efforts.

## Figures and Tables

**Table 1:** Summary statistics for the TCM gene network and GWAS disease-gene network.

TCM gene network			
Number of compounds	Number of TCM-target genes	Average number of genes per TCM compound	Number of TCM-gene interactions
102	7380	430	43,839

GWAS disease-gene network			
Number of diseases	Number of disease genes	Average number of genes per disease	Number of disease-gene interactions
293	7417	134	39,262

**Table 2:** GWAS disease-TCM gene pairs with *q* < 0.2 identified from this study.

TCM name	Diseases (*Z*-score)
1*β*-Hydroxyalantolactone	Ovarian cysts (3.952), ovarian diseases (3.365)
Ainsliadimer A	Neoplasms, glandular and epithelial (4.051)
Artemisinin	Diabetes mellitus, type 1 (4.526)
Benzoylaconitine	Embolism and thrombosis (5.058)
Benzyl benzoate	Embolism and thrombosis (6.157)
Berberine hydrochloride	Central nervous system infections (4.626) hemorrhage (5.090), hypertension, pregnancy-induced (4.347), prion diseases (5.433)
Borneol	Embolism and thrombosis (4.754)
Britanin	Central nervous system infections (5.323), prion diseases (4.554)
Bruceine D	Intestinal diseases (3.126), lip diseases (4.480), lymphoproliferative disorders (3.190), mouth abnormalities (4.471), skin diseases, papulosquamous (4.167)
Bufalin	Lip diseases (12.748), mouth abnormalities (12.730), skin diseases, papulosquamous (6.010), spinal diseases (5.987)
Chelerythrine	Autoimmune diseases of the nervous system (3.526)
Cholic acid	Embolism and thrombosis (4.536)
Cinnamaldehyde	Embolism and thrombosis (4.559)
Cinnamic acid	Embolism and thrombosis (5.398)
Curculigoside	Hypersensitivity, immediate (3.676)
Daidzin	Autoimmune diseases of the nervous system (4.358), bone marrow diseases (5.015), demyelinating autoimmune diseases, cns (3.744)
Deoxycholic acid	Embolism and thrombosis (5.072)
Four mixture	Autoimmune diseases of the nervous system (3.198), breast diseases (4.159), breast neoplasms (4.137), demyelinating autoimmune diseases, cns (3.263), gastrointestinal neoplasms (3.648), lymphoproliferative disorders (4.073), neoplasms, glandular and epithelial (3.244)
Gamabufatalin	Gastroenteritis (4.344), skin diseases, papulosquamous (5.028)
Gentiopicroside	Breast diseases (4.249), breast neoplasms (4.251)
Ginsenoside Rb2	Embolism and thrombosis (4.604)
Ginsenoside Rb3	Embolism and thrombosis (5.758)
Ginsenoside Rc	Embolism and thrombosis (6.291)
Ginsenoside Rd	Embolism and thrombosis (5.214)
Ginsenoside Re	Embolism and thrombosis (5.722)
Ginsenoside Rg1	Skin diseases, papulosquamous (4.279)
Isoborneol	Embolism and thrombosis (4.441)
Japonicone A	Acneiform eruptions (3.354), arthritis, rheumatoid (3.591), breast diseases (4.482), breast neoplasms (4.473), central nervous system infections (4.303), cocaine-related disorders (3.625), digestive system neoplasms (3.370), gastrointestinal neoplasms (5.216), intestinal diseases (3.592), leukemia (3.687), lymphoma (4.369), lymphoproliferative disorders (4.066), neoplasms, glandular and epithelial (3.772), ovarian diseases (3.535), prion diseases (5.863), rheumatic diseases (3.599), sebaceous gland diseases (3.357), urinary bladder diseases (3.722), urologic neoplasms (3.506)
Liquiritin	Autoimmune diseases of the nervous system (3.704), glucose metabolism disorders (4.245), hypersensitivity, immediate (3.993)
Narciclasine	Bone marrow diseases (3.974), gastroenteritis (3.703), intestinal diseases (3.734), lymphoma (3.848), lymphoproliferative disorders (4.190), osteitis deformans (3.623), peripheral vascular diseases (3.707), prion diseases (4.772), skin diseases, papulosquamous (4.086)
Nitidine chloride	Arthritis (3.319), bone marrow diseases (3.701), breast diseases (5.287), breast neoplasms (5.259), diabetes mellitus, type 2 (5.288), digestive system neoplasms (3.605), gastrointestinal neoplasms (3.712), glucose metabolism disorders (5.115), intestinal diseases (3.572), lymphoproliferative disorders (3.936), neoplasms, glandular and epithelial (4.427)
Notoginsenoside R1	Ovarian cysts (4.713), ovarian diseases (4.526)
Oleanic acid	Breast diseases (3.698), breast neoplasms (3.681), digestive system neoplasms (3.838), gastrointestinal neoplasms (4.394), muscular diseases (4.916), myositis (4.915), neoplasms (5.028)
Osthole	Neoplasms (5.137)
Salidroside	Autoimmune diseases of the nervous system (3.615), hypersensitivity, immediate (3.817), neoplasms (4.994)
Sanguinarine	Breast diseases (4.521), breast neoplasms (4.535)
Santonin	Breast neoplasms (3.506), gastrointestinal neoplasms (3.576)
Schisantherin A	Hypersensitivity, immediate (3.660)
Tanshinone IIA	Body weight (3.665), central nervous system infections (4.831), prion diseases (5.608)
Tetrahydropalmatine	Autoimmune diseases of the nervous system (4.315), demyelinating autoimmune diseases, cns (3.999), hypersensitivity, immediate (3.792)

## Data Availability

The web application is freely available at http://tanlab.ucdenver.edu/TCMHub.

## Supplementary Materials

Supplementary Materials Supplementary Table 1: list of the 102 TCM used in this study.
