# Supplementary material for: Systems Pharmacology-Based Approach of Connecting Disease Genes in Genome-Wide Association Studies with Traditional Chinese Medicine
Source: Int J Genomics. 2018 Mar 22;2018:7697356. doi: 10.1155/2018/7697356 (PMC5885494; doi:10.1155/2018/7697356)
Supplement: Supplementary Materials — Supplementary Table 1: list of the 102 TCM used in this study. [file 7697356.f1.pdf]

SUPPLEMENTARY TABLE 1. List of the 102 TCM used in this study.

No.	TCM Component	No.	TCM Component
1	(+)2-(1-hydroxyl-4-oxocyclohexyl) ethyl caffeate	52	Ginsenoside Rg1
2	1β-hydroxyalantolactone	53	Glycyrrhizic acid
3	6-gingerol	54	Hesperidin
4	Aconitine	55	Honokiol
5	Acteoside	56	Hydroxysafflor yellow A
6	Ainsliadimer A	57	Hyodeoxycholic acid
7	Alantolactone	58	Hypaconitine
8	Andrographolide	59	Hyperoside
9	Anhydroicaritin	60	Imperatorin
10	Arenobufagin	61	Isoalantolactone
11	Artemisinin	62	Isoborneol
12	Astragaloside IV	63	Japonicone A
13	Bacopaside I	64	L-scopolamine
14	Benzoylaconitine	65	Liquiritin
15	Benzoylhypaconitine	66	Lobetyolin
16	Benzyl benzoate	67	Macrozamin
17	Berberine hydrochloride	68	Magnolol
18	Bilobalide	69	Matrine
19	Borneol	70	Muscone
20	Britanin	71	Narciclasine
21	Bruceine D	72	Nitidine chloride
22	Bufalin	73	Notoginsenoside R1
23	Bufotaline	74	Oleanic acid
24	Chelerythrine	75	Oridonin
25	Chenodeoxycholic acid	76	Osthole
26	Chlorogenic acid	77	Oxymatrine
27	Cholic acid	78	Paeoniflorin
28	Cinnamaldehyde	79	Phillyrin
29	Cinnamic acid	80	Protocatechuic aldehyde
30	Cinobufogenin	81	Puerarin
31	Cinobufotalin	82	Resibufogenin
32	Curculigoside	83	Resveratrol
33	Daidzin	84	Saikosaponin A
34	Deoxycholic acid	85	Saikosaponin D
35	Dioscin	86	Salidroside
36	Emodin	87	Salvianic acid A sodium
37	Ephedrine hydrochloride	88	Salvianolic acid B
38	Ferulic acid	89	Sanguinarine
39	Four mixture	90	Santonin
40	Gallic acid	91	Schisantherin A
41	Gamabufatalin	92	Schizandrin
42	Gastrodin	93	Scutellarein
43	Geniposide	94	Sennoside A
44	Gentiopicroside	95	Silybin
45	Ginkgolide B	96	Stachydrine hydrochloride
46	Ginsenoside Rb1	97	Strychnine
47	Ginsenoside Rb2	98	Tanshinone IIA
48	Ginsenoside Rb3	99	Telocinobufagin
49	Ginsenoside Rc	100	Tetrahydropalmatine
50	Ginsenoside Rd	101	Ursodeoxycholic acid
51	Ginsenoside Re	102	β-ecdysterone
